# Prediction of Antibiotic Resistance in Patients With a Urinary Tract Infection: Algorithm Development and Validation

**DOI:** 10.2196/51326

**Published:** 2024-02-29

**Authors:** Nevruz İlhanlı, Se Yoon Park, Jaewoong Kim, Jee An Ryu, Ahmet Yardımcı, Dukyong Yoon

**Affiliations:** 1 Department of Biomedical Systems Informatics Yonsei University College of Medicine Yongin Republic of Korea; 2 Department of Biostatistics and Medical Informatics Akdeniz University Antalya Turkey; 3 Department of Hospital Medicine Yongin Severance Hospital Yonsei University College of Medicine Yongin Republic of Korea; 4 Center for Digital Health Yongin Severance Hospital Yonsei University Health System Yongin Republic of Korea; 5 Institute for Innovation in Digital Healthcare Severance Hospital Seoul Republic of Korea

**Keywords:** antibiotic resistance, machine learning, urinary tract infections, UTI, decision support

## Abstract

**Background:**

The early prediction of antibiotic resistance in patients with a urinary tract infection (UTI) is important to guide appropriate antibiotic therapy selection.

**Objective:**

In this study, we aimed to predict antibiotic resistance in patients with a UTI. Additionally, we aimed to interpret the machine learning models we developed.

**Methods:**

The electronic medical records of patients who were admitted to Yongin Severance Hospital, South Korea were used. A total of 71 features extracted from patients’ admission, diagnosis, prescription, and microbiology records were used for classification. UTI pathogens were classified as either sensitive or resistant to cephalosporin, piperacillin-tazobactam (TZP), carbapenem, trimethoprim-sulfamethoxazole (TMP-SMX), and fluoroquinolone. To analyze how each variable contributed to the machine learning model’s predictions of antibiotic resistance, we used the Shapley Additive Explanations method. Finally, a prototype machine learning–based clinical decision support system was proposed to provide clinicians the resistance probabilities for each antibiotic.

**Results:**

The data set included 3535, 737, 708, 1582, and 1365 samples for cephalosporin, TZP, TMP-SMX, fluoroquinolone, and carbapenem resistance prediction models, respectively. The area under the receiver operating characteristic curve values of the random forest models were 0.777 (95% CI 0.775-0.779), 0.864 (95% CI 0.862-0.867), 0.877 (95% CI 0.874-0.880), 0.881 (95% CI 0.879-0.882), and 0.884 (95% CI 0.884-0.885) in the training set and 0.638 (95% CI 0.635-0.642), 0.630 (95% CI 0.626-0.634), 0.665 (95% CI 0.659-0.671), 0.670 (95% CI 0.666-0.673), and 0.721 (95% CI 0.718-0.724) in the test set for predicting resistance to cephalosporin, TZP, carbapenem, TMP-SMX, and fluoroquinolone, respectively. The number of previous visits, first culture after admission, chronic lower respiratory diseases, administration of drugs before infection, and exposure time to these drugs were found to be important variables for predicting antibiotic resistance.

**Conclusions:**

The study results demonstrated the potential of machine learning to predict antibiotic resistance in patients with a UTI. Machine learning can assist clinicians in making decisions regarding the selection of appropriate antibiotic therapy in patients with a UTI.

## Introduction

Urinary tract infection (UTI) refers to an infection that occurs in any part of the urinary system, including the kidneys, ureters, urinary bladder, urethra, and other auxiliary structures [1,2]. Globally, UTIs are the most prevalent type of infectious disease, with around 150-250 million cases occurring each year [3]. Considerable morbidity and mortality result from these infections [4]. Typically, the most effective treatment for UTIs is the administration of antibiotics [3]. However, inappropriate use of antibiotics can permanently affect the normal microbiota of the urinary tract system and lead to antibiotic resistance [5].

The antibiotic susceptibility test is commonly used to identify antibiotic resistance, but it takes 24-48 hours to obtain test results [6,7]. However, in the clinical workflow, clinicians need to identify antibiotic resistance quickly to provide effective treatment for patients with UTIs. For this reason, early prediction of antibiotic resistance in patients with UTIs is important to guide the selection of appropriate antibiotic therapy. Machine learning can be used to develop prediction models and clinical decision support systems (CDSSs) to identify antibiotic resistance and support the selection of appropriate antibiotic therapy for patients with a UTI.

Several efforts have been made to predict antibiotic resistance in patients with UTIs using data from patients’ electronic medical records (EMRs), including demographics, prescriptions, comorbidities, procedures, and laboratory tests. These investigations have yielded promising results. Some of these studies were limited to specific patient groups, including patients with uncomplicated UTIs [8] and patients treated in the emergency department [9]. In other studies, researchers worked with heterogeneous data that were not limited to a specific patient group [10-12]. However, prior studies that analyzed heterogeneous data did not address the interpretation of machine learning models. The black-box nature of machine learning is a limiting factor not only in its use for antibiotic resistance prediction but also in its wider clinical use [13,14]. Thus, interpreting the results obtained by the machine learning model is crucial in increasing users’ trust in the machine learning model [15,16]. Furthermore, these studies did not address the development of the CDSS with the prediction models they built.

In this study, we aimed to predict antibiotic resistance in patients with a UTI. Heterogeneous data that were not limited to a specific patient group were used. UTI pathogens were classified as either sensitive or resistant to 5 commonly used antibiotics in UTI treatment: cephalosporin, piperacillin-tazobactam (TZP), carbapenem, trimethoprim-sulfamethoxazole (TMP-SMX), and fluoroquinolone. In addition, our objective was to understand and explain the inner workings of the machine learning models we developed. Eventually, a prototype CDSS was developed to provide clinicians the resistance probabilities for each antibiotic.

## Methods

### Ethical Considerations

Ethics approval for the study was obtained from the institutional review board of Yonsei University Severance Hospital on June 6, 2022 (approval 9-2023-0095). The informed consent was not required due to the retrospective nature of the study.

### Data Set Description and Study Design

**Figure 1:**
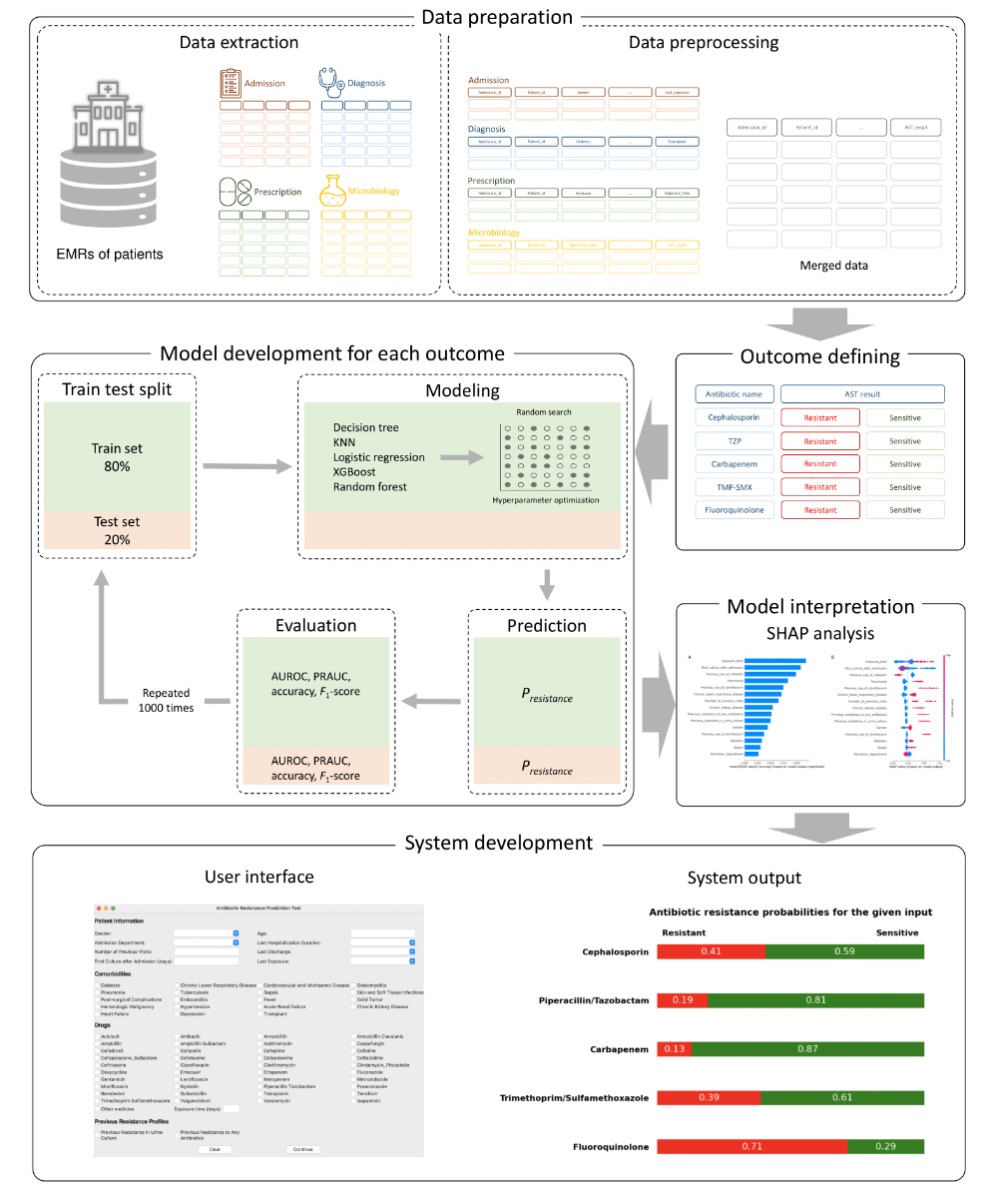
Summary of the research process. AST: antibiotic susceptibility test; AUROC: area under the receiver operating characteristic curve; EMR: electronic medical record; KNN: k-nearest neighbor; PRAUC: precision-recall area under the curve; SHAP: Shapley Additive Explanations; TMP-SMX: trimethoprim-sulfamethoxazole; TZP: piperacillin-tazobactam; XGBoost: Extreme Gradient Boosting.

In this study, we used the EMRs of patients who were admitted to Yongin Severance Hospital, South Korea, between October 2012 and October 2022. To build the prediction models, admission, diagnosis, prescription, and microbiology records were extracted. The summary of the research process is presented in Figure 1.

### Data Preprocessing

The microbiology table contained 143,114 urine cultures collected from 6011 patients during 7719 admissions. Since positive samples typically indicate the presence of bacteriuria, and urine culture samples were typically collected from patients with UTI symptoms, we considered these to be indicative of a UTI [10]. The resistance profiles were evaluated based on the Clinical and Laboratory Standards Institute guidelines, where intermediate-level resistance was considered sensitive. To assess the resistance of UTI pathogens to antibiotic classes, antibiotics were grouped as cephalosporin, TZP, carbapenem, TMP-SMX, and fluoroquinolone. The antibiotics included in each antibiotic class are presented in Multimedia Appendix 1. The patients’ demographic information was extracted from the admission table, their comorbidities were extracted from the diagnosis table, and their drug use information was extracted from the prescription table. For all input variables, the time of the first culture test was considered as the end point, and only data collected before the first culture test were used. After preprocessing and variable extraction from the raw data, the tables were combined using the admission number as the primary key. Missing data were excluded from the study. Patients aged 19 years and older and 100 years and younger at admission were included in the study, and numerical variables were standardized. A total of 71 features were used to classify UTI pathogens as either sensitive or resistant to each antibiotic. The predictors for the prediction models were selected by considering related works and using clinical judgment. Additionally, the threshold values for binarization were selected according to the literature [17] and the expert assessment of a specialist in infectious diseases. Detailed information about the predictors can be found in Multimedia Appendix 2.

### Machine Learning Model Development

We used a repeated train test split approach for modeling. The data sets were split into training and test sets using an 80:20 ratio, and the training sets were used for the development of the machine learning models. When splitting the data into training and test sets, data points from the same patient and admission were exclusively included in either the training or test data set to prevent potential data leakage and ensure the models were evaluated on previously unseen data. At each iteration, we created different training and test data sets by changing the random seed. Decision tree, k-nearest neighbor, logistic regression, Extreme Gradient Boosting, and random forest were used for modeling. The hyperparameters of the machine learning models were optimized by using the random search hyperparameter optimization method with 10-fold cross-validation on the training data set. We stored the performance of the prediction models at each iteration, and the mean of performance metrics was calculated. The procedure of splitting the data, optimizing hyperparameters, modeling, and evaluation was iteratively repeated 1000 times to classify UTI pathogens as either sensitive or resistant to cephalosporin, TZP, carbapenem, TMP-SMX, and fluoroquinolone. The machine learning models were built using Python (version 3.10.4; Python Software Foundation).

### Machine Learning Model Interpretation

To analyze the contribution of the variables to the machine learning models in predicting antibiotic resistance, we used the Shapley Additive Explanations (SHAP) method. The SHAP values of the random forest models that showed superior performance compared to other machine learning methods were evaluated. The random forest model with the highest area under the receiver operating characteristic curve (AUROC) on the test set across all iterations for each antibiotic was used for SHAP analysis. Python (version 3.10.4; Python Software Foundation) was used for SHAP analysis.

### CDSS Development

To develop the CDSS prototype, the random forest model with the highest AUROC on the test set across all iterations for each antibiotic was used. The CDSS prototype was developed using the *tkinter* package in Python (version 3.10.4; Python Software Foundation).

### Evaluation

The performance of the machine learning model for predicting antibiotic resistance was evaluated on the training and test sets using the AUROC with 95% CIs, precision-recall area under the curve (PRAUC), accuracy, and *F*_1_-score performance metrics. Herein, the AUROC value was considered the main evaluation metric. The definitions of the performance metrics we used are provided below.

- AUROC: The AUROC is a widely used metric that represents a classifier’s ability to discriminate between positive instances and negative instances [18].
- PRAUC: PRAUC refers to the area under the precision-recall curve that plots precision as a function of recall for all the possible decision thresholds [19].
- Accuracy: Accuracy is the ratio of correctly classified samples to all samples.

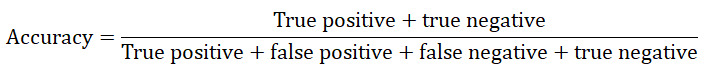

- F_1_-score: F_1_-score is the harmonic mean of precision and recall metrics.

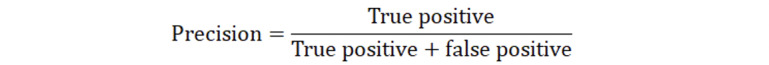





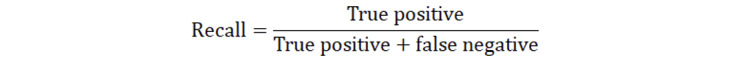





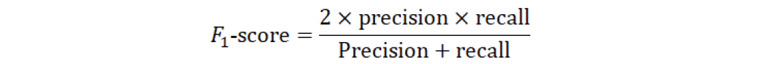



Python (version 3.10.4; Python Software Foundation) was used to evaluate the prediction models.

## Results

### Data Set Characteristics

The general characteristics of the data set used in this study are presented in Table 1. The data set included 3535, 737, 708, 1582, and 1365 samples for cephalosporin, TZP, TMP-SMX, fluoroquinolone, and carbapenem resistance prediction models, respectively. *Escherichia coli* was the most frequently isolated bacterial specimen across all antibiotics.

**Table 1:** General characteristics of the data set.

	Cephalosporin	TZP^a^	TMP-SMX^b^	Fluoroquinolone	Carbapenem
Samples, n	3535	737	708	1582	1365
Admissions, n	396	366	374	571	392
Patients, n	390	360	368	557	386
Resistance, n (%)	1492 (42.2)	169 (22.9)	281 (39.7)	1014 (64.1)	142 (10.4)
Age (years), mean (SD)	71.5 (14.4)	71.4 (14.4)	71.4 (14.4)	71.9 (14.4)	71.7 (14.3)
Female, n (%)	2597 (73.5)	523 (71)	507 (71.6)	1013 (64)	994 (72.8)
Most common bacteria (*Escherichia coli*), n (%)	1650 (46.7)	312 (42.3)	331 (46.7)	349 (22)	624 (45.7)
Second-most common bacteria (*Klebsiella pneumoniae*), n (%)	556 (15.7)	109 (14.8)	111 (15.7)	305 (19.3)^c^	220 (16.1)
Third-most common bacteria (*Pseudomonas aeruginosa*), n (%)	168 (4.7)	69 (9.4)	21 (3)^d^	180 (11.4)^e^	83 (6.1)

^a^TZP: piperacillin-tazobactam.

^b^TMP-SMX: trimethoprim-sulfamethoxazole.

^c^The isolated bacterial specimen is *Enterococcus faecium*.

^d^The isolated bacterial specimen is *Citrobacter freundii*.

^e^The isolated bacterial specimen is *Enterococcus faecalis*.

### Model Performance

The performance analysis of the random forest models is presented in Table 2. The AUROC values were 0.777 (95% CI 0.775-0.779), 0.864 (95% CI 0.862-0.867), 0.877 (95% CI 0.874-0.880), 0.881 (95% CI 0.879-0.882), and 0.884 (95% CI 0.884-0.885) in the training set and 0.638 (95% CI 0.635-0.642), 0.630 (95% CI 0.626-0.634), 0.665 (95% CI 0.659-0.671), 0.670 (95% CI 0.666-0.673), and 0.721 (95% CI 0.718-0.724) in the test set for predicting resistance to cephalosporin, TZP, carbapenem, TMP-SMX, and fluoroquinolone, respectively. The performance analysis of the other machine learning models is presented in Multimedia Appendices 3-6.

**Table 2:** Classification performances of the random forest models.

	Training set					Test set			
	AUROC^a^ (95% CI)	PRAUC^b^	Accuracy	*F*_1_-score	AUROC (95% CI)		PRAUC	Accuracy	*F*_1_-score
Cephalosporin	0.777 (0.775-0.779)	0.725	0.715	0.676	0.638 (0.635-0.642)		0.547	0.603	0.556
TZP^c^	0.864 (0.862-0.867)	0.688	0.808	0.652	0.630 (0.626-0.634)		0.332	0.641	0.313
Carbapenem	0.877 (0.874-0.880)	0.539	0.822	0.493	0.665 (0.659-0.671)		0.222	0.725	0.220
TMP-SMX^d^	0.881 (0.879-0.882)	0.829	0.822	0.781	0.670 (0.666-0.673)		0.568	0.638	0.560
Fluoroquinolone	0.884 (0.884-0.885)	0.938	0.802	0.832	0.721 (0.718-0.724)		0.813	0.657	0.706

^a^AUROC: area under the receiver operating characteristic curve.

^b^PRAUC: precision-recall area under the curve.

^c^TZP: piperacillin-tazobactam.

^d^TMP-SMX: trimethoprim-sulfamethoxazole.

### Important Features

**Figure 2:**
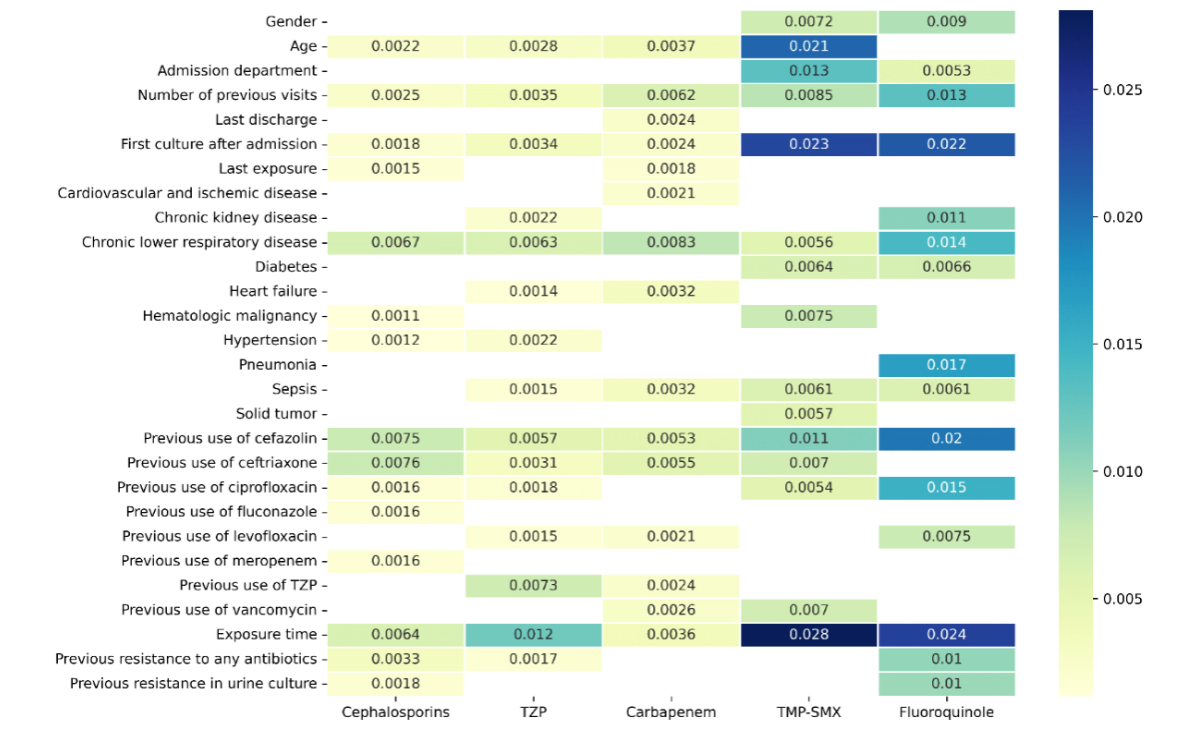
SHAP values of the 15 most important features in the prediction models. SHAP: Shapley Additive Explanations; TMP-SMX: trimethoprim-sulfamethoxazole; TZP: piperacillin-tazobactam.

The SHAP values of the 15 most important features in the random forest models are presented in Figure 2.

**Figure 3:**
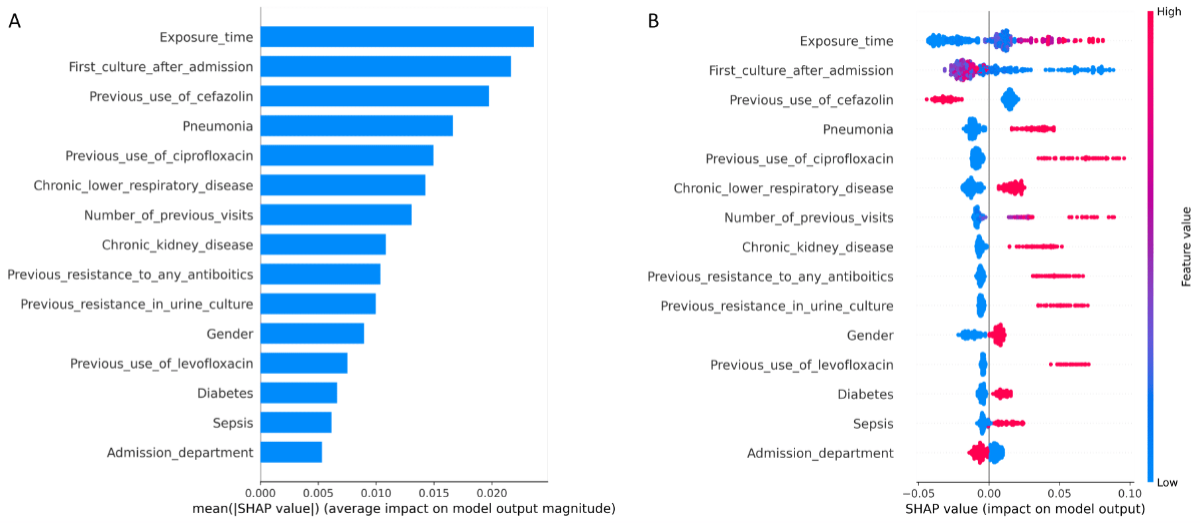
SHAP analysis results of fluoroquinolone resistance prediction model. (A) The feature importance bar plot. (B) The SHAP summary dot plot. SHAP: Shapley Additive Explanations.

The SHAP feature importance bar plot (Figure 3A) and SHAP summary plot (Figure 3B) of the fluoroquinolone resistance prediction model are presented in Figure 3. The SHAP feature importance plot and SHAP summary plot of the other antibiotic prediction models are presented in Multimedia Appendices 7-10.

### Clinical Decision Support System

**Figure 4:**
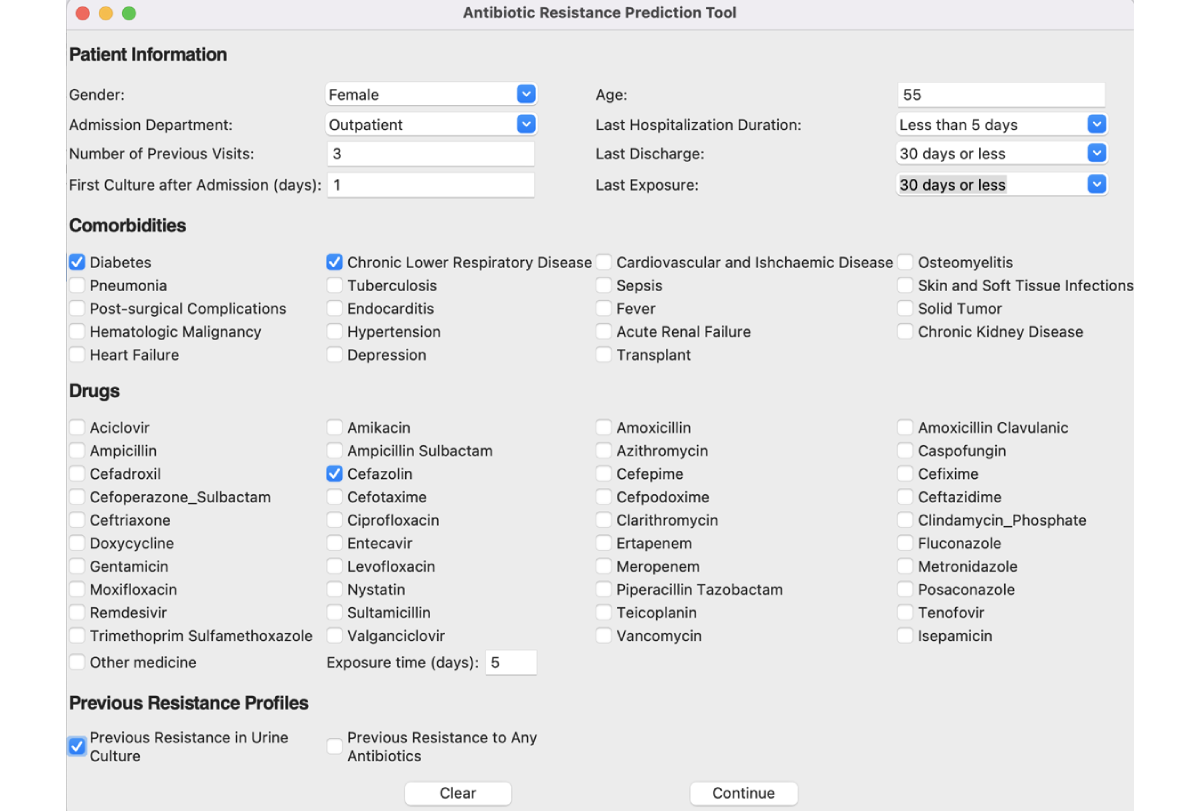
The user interface of the clinical decision support system.

The user interface of the CDSS is shown in Figure 4. The CDSS prototype obtains data from the user and produces antibiotic resistance probabilities for each antibiotic.

We presented the CDSS prototype on a scenario. In this case, a female aged 55 years was admitted to the hospital’s outpatient department. The patient previously visited the hospital 3 times and was readmitted to the hospital within 30 days of her last 3-day stay. The duration between the patient’s admission to the hospital and the first culture was 1 day. The patient was previously diagnosed with diabetes and chronic lower respiratory disease. Additionally, the patient had a history of cefazolin use in the last 30 days and resistance in urine culture.

**Figure 5:**
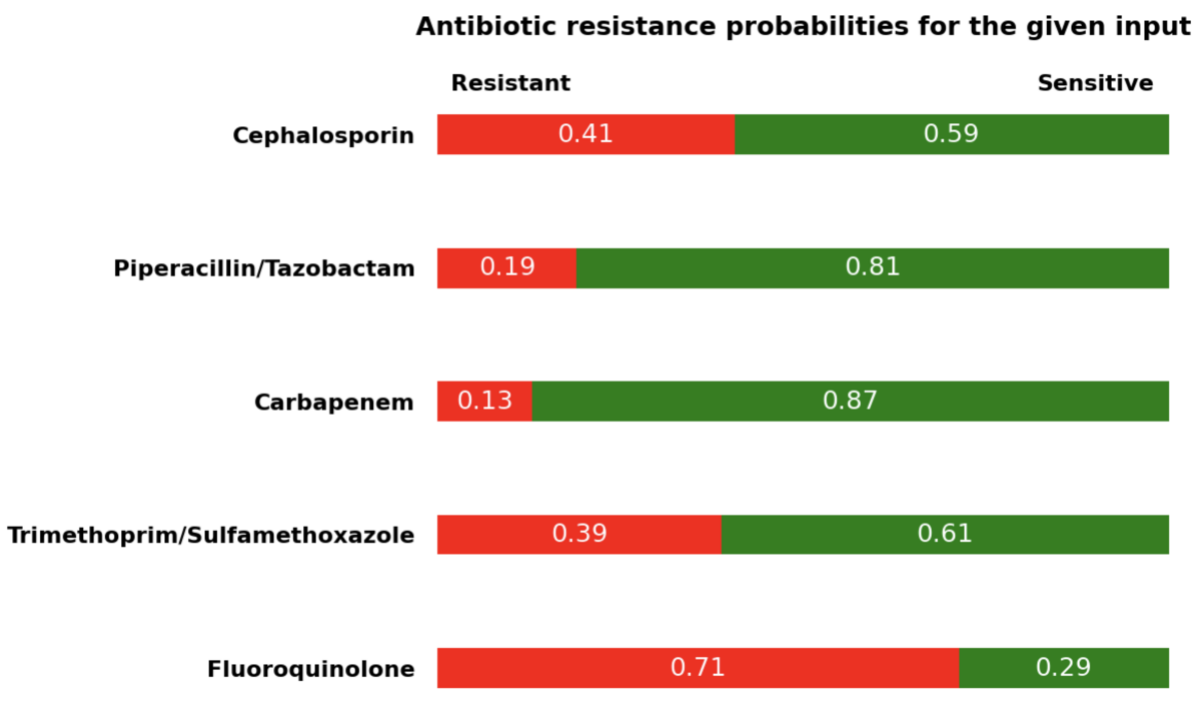
The screenshot of system output for the given data.

The system output for the given scenario is shown in Figure 5. The system produced resistance probabilities for each antibiotic. For the given scenario, the system produced a 71% probability of fluoroquinolone resistance, a 41% probability of cephalosporin resistance, a 39% probability of TMP-SMX resistance, a 19% probability of TZP resistance, and a 13% probability of carbapenem resistance.

## Discussion

### Principal Findings

In this study, our main objective was to predict cephalosporin, TZP, carbapenem, TMP-SMX, and fluoroquinolone resistance in patients with UTI and develop a CDSS with the machine learning models we built. Moreover, we identified the most important features for predicting antibiotic resistance in patients with UTI using SHAP analysis.

Our prediction models achieved AUROCs of 0.777 (95% CI 0.775-0.779), 0.864 (95% CI 0.862-0.867), 0.877 (95% CI 0.874-0.880), 0.881 (95% CI 0.879-0.882), and 0.884 (95% CI 0.884-0.885) in the training set and 0.638 (95% CI 0.635-0.642), 0.630 (95% CI 0.626-0.634), 0.665 (95% CI 0.659-0.671), 0.670 (95% CI 0.666-0.673), and 0.721 (95% CI 0.718-0.724) in the test set for predicting resistance to cephalosporin, TZP, carbapenem, TMP-SMX, and fluoroquinolone, respectively. The fluoroquinolone resistance prediction model showed superior performance, as confirmed by its high AUROC values in both the training and test sets. On the other hand, the cephalosporin resistance prediction model showed poor performance, as confirmed by the low AUROC values in both training and test sets.

According to SHAP analysis, the contribution of the variables varied for each antibiotic; however, we found that the number of previous visits, first culture after admission, chronic lower respiratory diseases, administration of drugs before infection, and exposure time to these drugs were important predictors across all antibiotics. Factors such as the first culture after admission, exposure time, and the number of previous visits were found to affect resistance, which can be explained by the impact of health care–associated infections. Chronic lower respiratory and kidney diseases are also likely to be associated with frequent visits to health care facilities, although it is difficult to confirm the actual number of visits. However, this suggests that the characteristics of health care–seeking behavior in patients with specific underlying diseases may influence resistance [20]. Interestingly, the use of cefazolin had a negative impact on the development of resistance for all antibiotics. This is because cefazolin is one of the narrow-spectrum antibiotics used in less severe patients. Further research is needed to examine these results.

### Comparison to Prior Work

Past efforts to predict antibiotic resistance in patients with UTIs have had promising results, with the lowest AUROC being 0.58 for predicting TMP-SMX resistance [12] and the highest AUROC being 0.83 for predicting ciprofloxacin resistance [9]. In comparison, our prediction models demonstrated comparable performance to these prior works. Some previous studies on predicting antibiotic resistance in patients with UTIs were limited to specific patient groups, including patients with uncomplicated UTIs [8] and patients treated in the emergency department [9]. We analyzed heterogeneous data that were not limited to a specific patient group or bacteria. This approach provides a more comprehensive insight into the prediction of antibiotic resistance in patients with UTIs. Similarly, Lewin-Epstein et al [21] analyzed heterogeneous data and were able to achieve AUROC values ranging from 0.73 to 0.79 for the prediction of ceftazidime, gentamicin, imipenem, ofloxacin, and TMP-SMX resistance. Their data contained multiple culture tests, which provided a more comprehensive approach to predicting antibiotic resistance. Although urine cultures can be used to infer colonized resistance in patients, further research is needed to extend culture results beyond urine.

### Limitations

While this study provides insights into predicting antibiotic resistance in patients with UTIs, it has some limitations. First, this study is the lack of multidrug resistance classification. The data set we used in this study did not contain a sufficient amount of multidrug resistance outcomes to build a classification model for the prediction of multidrug resistance. Furthermore, our prediction models were developed using prescription records within the hospital setting. However, patients may have used antibiotics outside of the hospital setting during visits to other hospitals. The lack of information about past drug use could have negatively impacted the performance of our prediction models. To overcome this limitation, we intend to conduct further studies using data from the National Health Insurance Service of South Korea, which contain all past drug use information of the patients. Thus, we will have a more comprehensive data set. By using this approach, we may be able to develop more accurate machine learning models to predict antibiotic resistance and improve our ability to guide appropriate antibiotic therapy selection. Additionally, further development is required to address the limitations of prototype CDSS, including the integration of real-time patient data and validation in larger patient cohorts. Moreover, the prototype CDSS only gives the resistance risk probability to the user. However, a more comprehensive system that can provide decision support on the selection of appropriate therapy, dosage, and duration of treatment can be developed in further studies. Such a system has the potential to reduce the duration of treatment, number of antibiotics used, cost, mortality, and morbidity [22,23].

### Conclusions

In conclusion, our study results demonstrated that prediction models to predict antibiotic resistance in patients with UTIs can be constructed using routinely collected EMR data alone, without requiring additional laboratory tests or specialized tests. Machine learning techniques can be used to develop systems that can guide clinicians in selecting appropriate antibiotic therapy. This has the potential to prevent the risk of inappropriate antibiotic administration, thereby reducing patients’ risk of developing antibiotic resistance.

## Appendix

Multimedia Appendix 1 The antibiotics included in each antibiotic class.

Multimedia Appendix 2 Description of input variables.

Multimedia Appendix 3 Classification performances of the decision tree models.

Multimedia Appendix 4 Classification performances of the k-nearest neighbor models.

Multimedia Appendix 5 Classification performances of the logistic regression models.

Multimedia Appendix 6 Classification performances of the Extreme Gradient Boosting models.

Multimedia Appendix 7 SHAP analysis results of cephalosporin resistance prediction model. (A) The feature importance bar plot. (B) The SHAP summary dot plot. SHAP: Shapley Additive Explanations.

Multimedia Appendix 8 SHAP analysis results of TZP resistance prediction model. (A) The feature importance bar plot. (B) The SHAP summary dot plot. SHAP: Shapley Additive Explanations; TZP: piperacillin-tazobactam.

Multimedia Appendix 9 SHAP analysis results of carbapenem resistance prediction model. (A) The feature importance bar plot. (B) The SHAP summary dot plot. SHAP: Shapley Additive Explanations; TZP: piperacillin-tazobactam.

Multimedia Appendix 10 SHAP analysis results of TMP-SMX resistance prediction model. (A) The feature importance bar plot. (B) The SHAP summary dot plot. SHAP: Shapley Additive Explanations; TMP-SMX: trimethoprim-sulfamethoxazole.

## Abbreviations

AUROC: area under the receiver operating characteristic curve
CDSS: clinical decision support system
EMR: electronic medical record
PRAUC: precision-recall area under the curve
SHAP: Shapley Additive Explanations
TMP-SMX: trimethoprim-sulfamethoxazole
TZP: piperacillin-tazobactam
UTI: urinary tract infection
