# Supplementary material for: Prediction of Antibiotic Resistance in Patients With a Urinary Tract Infection: Algorithm Development and Validation
Source: JMIR Med Inform. 2024 Feb 29;12:e51326. doi: 10.2196/51326 (PMC10940975; doi:10.2196/51326)
Supplement: Multimedia Appendix 1 [file medinform_v12i1e51326_app1.docx]

**Cephalosporin:** Ceftazidime, Cefotaxime, Cefoxitin, Cefepime, Cefazolin, Cephalothin, Cefoperazone, Ceftriaxone

**Piperacillin-tazobactam (TZP):** TZP

**Trimethoprim-sulfamethoxazole (TMP-SMX):** TMP-SMX

**Fluoroquinolone:** Ciprofloxacin, Levofloxacin, Moxifloxacin

**Carbapenem:** Imipenem, Ertapenem
