# Supplementary material for: Prediction of Antibiotic Resistance in Patients With a Urinary Tract Infection: Algorithm Development and Validation
Source: JMIR Med Inform. 2024 Feb 29;12:e51326. doi: 10.2196/51326 (PMC10940975; doi:10.2196/51326)
Supplement: Multimedia Appendix 2 [file medinform_v12i1e51326_app2.docx]

**Gender:** A binary variable where 0 indicates male and 1 indicates female.

**Age:** The age of the patient during admission to the hospital.

**Admission department:** A binary variable where 0 indicates admission to an outpatient department and 1 indicates admission to an inpatient department.

**Last hospitalization duration:** The duration between the previous discharge date and previous admission date. A binary variable where 0 indicates less than 5 days and 1 indicates duration of 5 days or more.

**Number of previous visits:** The number of times the patient has been admitted to the hospital previously.

**Last discharge:** The time between the previous discharge date and current admission date. A binary variable where 0 indicates more than 30 days and 1 indicates a duration of 30 days or less.

**First culture after admission:** The time between the first culture date and admission date.

**Last exposure:** The time between drug stop date in previous hospital visit and current admission date. A binary variable where 0 indicates more than 30 days and 1 indicates a duration of 30 days or less.

**Comorbidities:** A set of binary variables indicating whether the patient has following comorbidities: diabetes, chronic lower respiratory disease, cardiovascular and ischemic disease, osteomyelitis, pneumonia, tuberculosis, sepsis, skin and soft tissue infections, post-surgical complications, endocarditis, fever, solid tumor, hematologic malignancy, hypertension, acute renal failure, chronic kidney disease, heart failure, depression, and transplant.

**Drugs:** A set of binary variables indicating whether a patient has administered a particular drug before first culture. This includes the top 40 most commonly prescribed drugs, as well as an additional category for all drugs taken that are not found in other categories. Each drug is represented by a binary variable, indicating whether it was administered or not.

**Exposure time:** The duration between the start and stop date of administered drugs.

**Previous resistance to any antibiotics:** A binary variable indicating whether the patient had a previous resistant culture to any antibiotic.

**Previous resistance in urine culture:** A binary variable indicating whether the patient had a previous resistant urine culture to any antibiotic.
