# Supplementary material for: Prediction of Antibiotic Resistance in Patients With a Urinary Tract Infection: Algorithm Development and Validation
Source: JMIR Med Inform. 2024 Feb 29;12:e51326. doi: 10.2196/51326 (PMC10940975; doi:10.2196/51326)
Supplement: Multimedia Appendix 3 [file medinform_v12i1e51326_app3.docx]

|  | Training Set | | | | Test Set | | | |
| --- | --- | --- | --- | --- | --- | --- | --- | --- |
|  | AUROC^a^ (95% CI) | PRAUC^b^ | Accuracy | F1 Score | AUROC^a^ (95% CI) | PRAUC^b^ | Accuracy | F1 Score |
| Cephalosporin | 0.667 (0.664 - 0.671) | 0.583 | 0.644 | 0.555 | 0.562 (0.558 - 0.566) | 0.470 | 0.573 | 0.444 |
| TZP^c^ | 0.676 (0.671 - 0.681) | 0.383 | 0.651 | 0.442 | 0.560 (0.554 - 0.566) | 0.275 | 0.600 | 0.320 |
| Carbapenem | 0.700 (0.695 - 0.705) | 0.259 | 0.743 | 0.329 | 0.549 (0.541 - 0.556) | 0.144 | 0.709 | 0.174 |
| TMP-SMX^d^ | 0.764 (0.759 - 0.769) | 0.692 | 0.735 | 0.636 | 0.552 (0.548 - 0.557) | 0.451 | 0.590 | 0.387 |
| Fluoroquinolone | 0.738 (0.732 - 0.744) | 0.813 | 0.666 | 0.684 | 0.617 (0.613 - 0.621) | 0.722 | 0.578 | 0.596 |

^a^AUROC: area under the ROC curve.

^b^PRAUC: precision-recall area under the curve.

^c^TZP: piperacillin-tazobactam.

^d^TMP-SMX: trimethoprim-sulfamethoxazole.
