# Supplementary material for: Prediction of Antibiotic Resistance in Patients With a Urinary Tract Infection: Algorithm Development and Validation
Source: JMIR Med Inform. 2024 Feb 29;12:e51326. doi: 10.2196/51326 (PMC10940975; doi:10.2196/51326)
Supplement: Multimedia Appendix 4 [file medinform_v12i1e51326_app4.docx]

|  | Training Set | | | | Test Set | | | |
| --- | --- | --- | --- | --- | --- | --- | --- | --- |
|  | AUROC^a^ (95% CI) | PRAUC^b^ | Accuracy | F1 Score | AUROC^a^ (95% CI) | PRAUC^b^ | Accuracy | F1 Score |
| Cephalosporin | 0.867 (0.867 - 0.868) | 0.788 | 0.787 | 0.754 | 0.547 (0.544 - 0.550) | 0.459 | 0.565 | 0.410 |
| TZP^c^ | 0.907 (0.906 - 0.907) | 0.707 | 0.783 | 0.663 | 0.523 (0.518 - 0.528) | 0.269 | 0.592 | 0.282 |
| Carbapenem | 0.941 (0.940 - 0.942) | 0.678 | 0.900 | 0.663 | 0.536 (0.532 - 0.540) | 0.129 | 0.783 | 0.163 |
| TMP-SMX^d^ | 0.882 (0.881 - 0.883) | 0.787 | 0.787 | 0.766 | 0.548 (0.544 - 0.553) | 0.464 | 0.539 | 0.468 |
| Fluoroquinolone | 0.929 (0.929 - 0.930) | 0.948 | 0.830 | 0.854 | 0.599 (0.596 - 0.602) | 0.705 | 0.568 | 0.612 |

^a^AUROC: area under the ROC curve.

^b^PRAUC: precision-recall area under the curve.

^c^TZP: piperacillin-tazobactam.

^d^TMP-SMX: trimethoprim-sulfamethoxazole.
