# Supplementary material for: Prediction of Antibiotic Resistance in Patients With a Urinary Tract Infection: Algorithm Development and Validation
Source: JMIR Med Inform. 2024 Feb 29;12:e51326. doi: 10.2196/51326 (PMC10940975; doi:10.2196/51326)
Supplement: Multimedia Appendix 5 [file medinform_v12i1e51326_app5.docx]

|  | Training Set | | | | Test Set | | | |
| --- | --- | --- | --- | --- | --- | --- | --- | --- |
|  | AUROC^a^ (95% CI) | PRAUC^b^ | Accuracy | F1 Score | AUROC^a^ (95% CI) | PRAUC^b^ | Accuracy | F1 Score |
| Cephalosporin | 0.718 (0.715 - 0.720) | 0.663 | 0.682 | 0.617 | 0.615 (0.611 - 0.619) | 0.537 | 0.595 | 0.497 |
| TZP^c^ | 0.615 (0.608 - 0.622) | 0.347 | 0.705 | 0.293 | 0.538 (0.533 - 0.542) | 0.274 | 0.659 | 0.194 |
| Carbapenem | 0.638 (0.630 - 0.646) | 0.216 | 0.769 | 0.204 | 0.565 (0.558 - 0.570) | 0.159 | 0.742 | 0.126 |
| TMP-SMX^d^ | 0.766 (0.762 - 0.770) | 0.712 | 0.735 | 0.653 | 0.633 (0.629 - 0.638) | 0.546 | 0.619 | 0.480 |
| Fluoroquinolone | 0.791 (0.787 - 0.794) | 0.874 | 0.727 | 0.758 | 0.704 (0.700 - 0.707) | 0.804 | 0.656 | 0.692 |

^a^AUROC: area under the ROC curve.

^b^PRAUC: precision-recall area under the curve.

^c^TZP: piperacillin-tazobactam.

^d^TMP-SMX: trimethoprim-sulfamethoxazole.
