# Supplementary material for: Prediction of Antibiotic Resistance in Patients With a Urinary Tract Infection: Algorithm Development and Validation
Source: JMIR Med Inform. 2024 Feb 29;12:e51326. doi: 10.2196/51326 (PMC10940975; doi:10.2196/51326)
Supplement: Multimedia Appendix 6 [file medinform_v12i1e51326_app6.docx]

|  | Training Set | | | | Test set | | | |
| --- | --- | --- | --- | --- | --- | --- | --- | --- |
|  | AUROC^a^ (95% CI) | PRAUC^b^ | Accuracy | F1 Score | AUROC^a^ (95% CI) | PRAUC^b^ | Accuracy | F1 Score |
| Cephalosporin | 0.846 (0.843 - 0.848) | 0.801 | 0.766 | 0.738 | 0.614 (0.611 - 0.617) | 0.526 | 0.584 | 0.514 |
| TZP^c^ | 0.887 (0.884 - 0.890) | 0.728 | 0.810 | 0.670 | 0.619 (0.614 - 0.623) | 0.333 | 0.648 | 0.319 |
| Carbapenem | 0.921 (0.919 - 0.923) | 0.640 | 0.862 | 0.571 | 0.636 (0.629 - 0.642) | 0.216 | 0.759 | 0.199 |
| TMP-SMX^d^ | 0.882 (0.879 - 0.885) | 0.830 | 0.818 | 0.780 | 0.611 (0.607 - 0.616) | 0.508 | 0.589 | 0.481 |
| Fluoroquinolone | 0.914 (0.912 - 0.917) | 0.951 | 0.838 | 0.865 | 0.694 (0.691 - 0.697) | 0.791 | 0.654 | 0.709 |

^a^AUROC: area under the ROC curve.

^b^PRAUC: precision-recall area under the curve.

^c^TZP: piperacillin-tazobactam.

^d^TMP-SMX: trimethoprim-sulfamethoxazole.
