# Supplementary figures and images for: Prediction of Antibiotic Resistance in Patients With a Urinary Tract Infection: Algorithm Development and Validation
Source: JMIR Med Inform. 2024 Feb 29;12:e51326. doi: 10.2196/51326 (PMC10940975; doi:10.2196/51326)

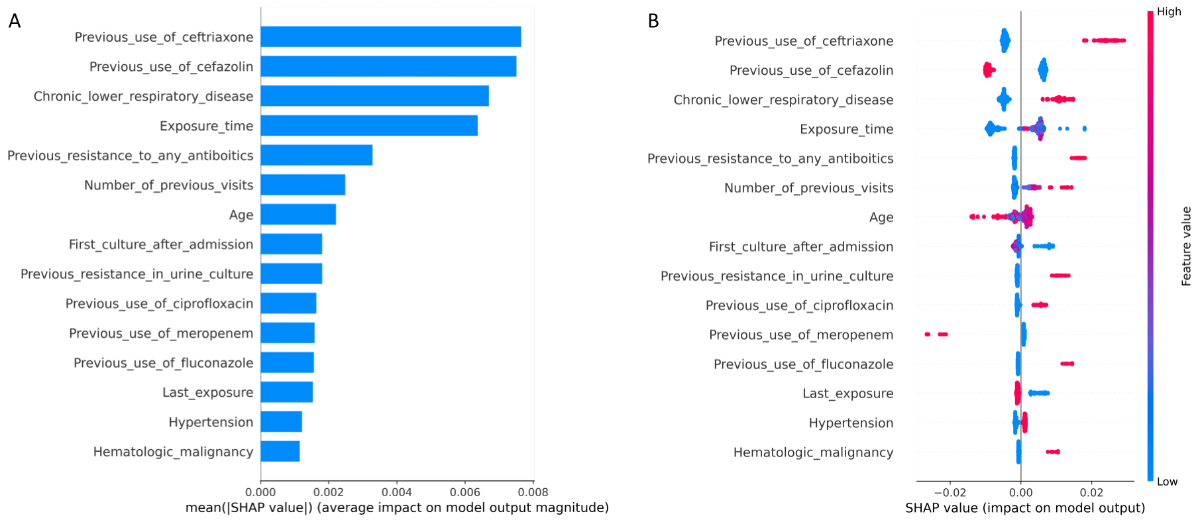

Supplement: Multimedia Appendix 7 [file medinform_v12i1e51326_app7.png]

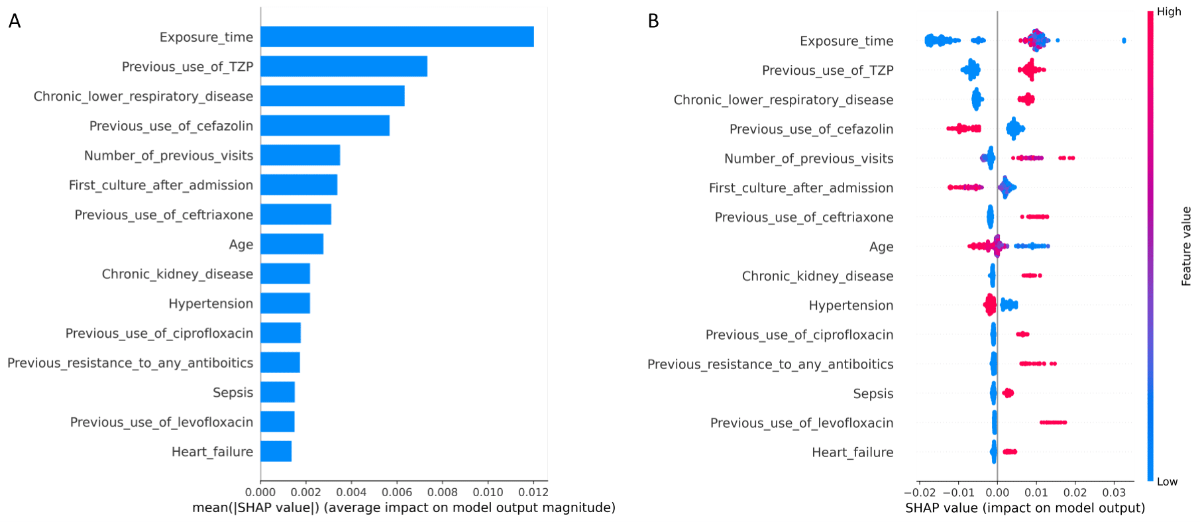

Supplement: Multimedia Appendix 8 [file medinform_v12i1e51326_app8.png]

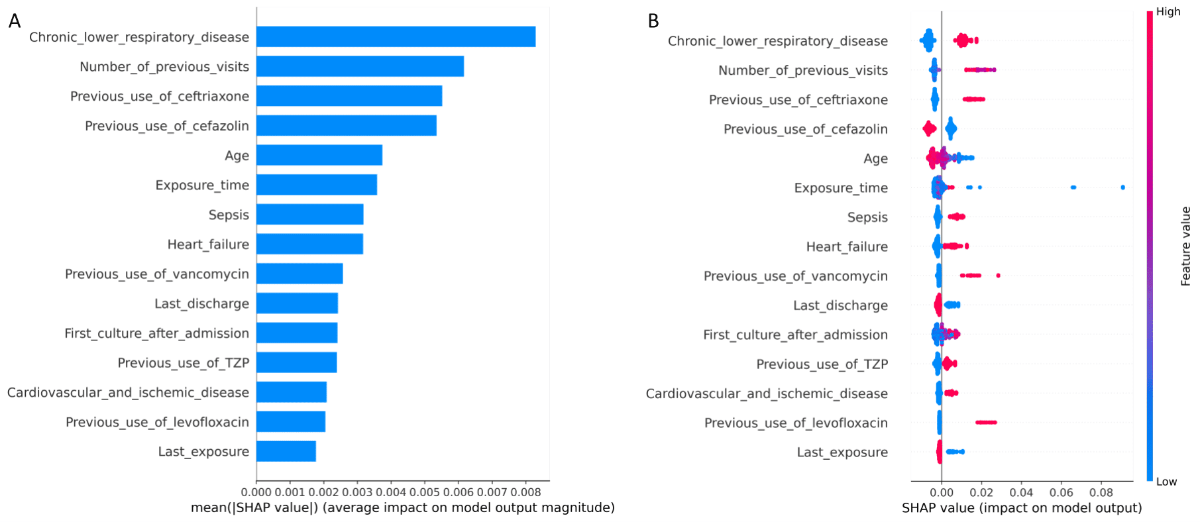

Supplement: Multimedia Appendix 9 [file medinform_v12i1e51326_app9.png]

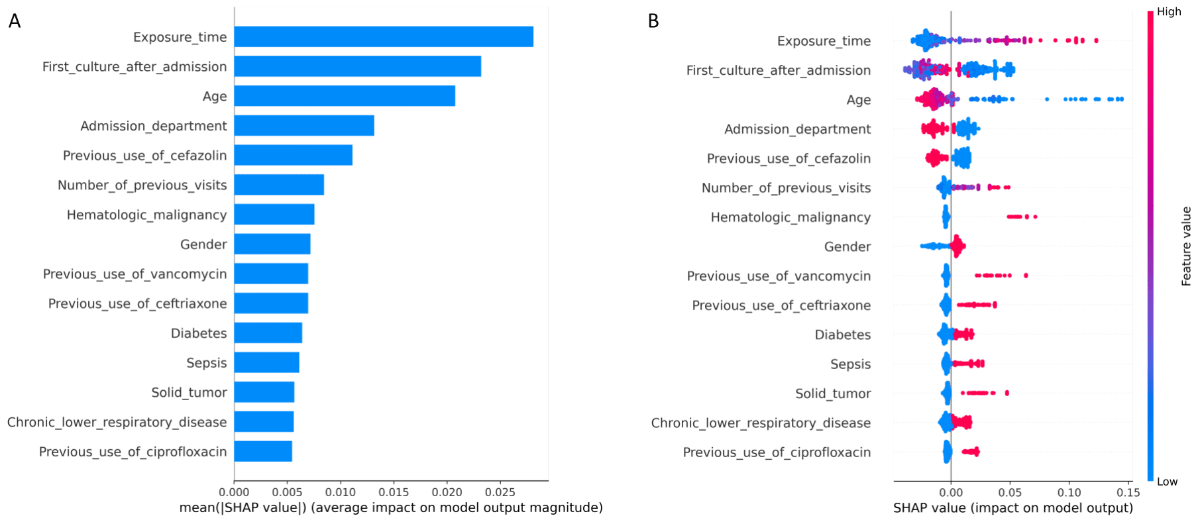

Supplement: Multimedia Appendix 10 [file medinform_v12i1e51326_app10.png]
